# Hypoxia-regulated components of the U4/U6.U5 tri-small nuclear riboprotein complex: possible role in autosomal dominant retinitis pigmentosa

**Published:** 2008-01-25

**Authors:** Rainald Schmidt-Kastner, Hideo Yamamoto, Duco Hamasaki, Hiroko Yamamoto, Jean-Marie Parel, Christoph Schmitz, C. Kathy Dorey, Janet C. Blanks, Markus N. Preising

**Affiliations:** 1 Charles E. Schmidt College of Biomedical Science, Florida Atlantic University (FAU), Boca Raton, FL; 2 Department of Psychiatry and Neuropsychology, Division of Cellular Neuroscience, Maastricht University, Maastricht, Netherlands; 3 Ophthalmic Biophysics Center, Bascom Palmer Eye Institute, University of Miami Miller School of Medicine, Miami, FL; 4 Department of Pediatric Ophthalmology, Strabismology; and Ophthalmogenetics, Regensburg University Medical Center, Regensburg, Germany; 5 Laboratory for Molecular Ophthalmology, Department of Ophthalmology, Universitaetsklinikum Giessen and Marburg GmbH, Giessen, Germany

## Abstract

**Purpose:**

High oxygen consumption and cyclical changes related to dark-adaptation are characteristic of the outer retina. Oxygenation changes may contribute to the selective vulnerability of the retina in retinitis pigmentosa (RP) patients, especially for those forms involving genes with global cellular functions. Genes coding for components of the U4/U6.U5 tri small nuclear ribonucleoprotein (tri-snRNP) complex of the spliceosome stand out, because mutations in four genes cause RP, i.e., RP9 (*PAP1*), RP11 (*PRPF31*), RP13 (*PRPF8*), and RP18 (*PRPF3*), while there is no degeneration outside the retina despite global expression of these genes. With the assumption that variable oxygenation plays a role in RP forms related to pre-mRNA splicing and the retina and brain are similar, we searched a data collection of ischemia-hypoxia regulated genes of the brain for oxygen regulated genes of the U4/U6.U5 tri-snRNP complex.

**Methods:**

A database of ischemia-hypoxia response (IHR) genes in the brain was generated from gene expression profiling studies [n=24]. Public databases (NCBI) were searched for RP genes with global function that are expressed in the brain. From the IHR gene list, we extracted genes that were directly related to retinal degeneration through a listed mutation (OMIM, Retnet, RISN). The database was then examined for indirect links to RP forms affecting the U4/U6.U5 tri-snRNP complex by searching for IHR genes contributing to this complex. Potential expression of matched genes in the retina was ascertained using NEIBank. Immunohistochemistry was used to localize a selected protein of the U4/U6.U5 tri-snRNP complex in cynomolgus monkey and human retina specimens.

**Results:**

The approach identified genes that cause retinal degeneration (*CNGB1, SEMA4A, RRG4*) or developmental changes (*SOX2*) when mutated. One IHR gene, *Pim1*, is the immediate binding partner for PAP1 (RP9). Three IHR genes linked the U4/U6.U5 tri-snRNP complex to regulation by oxygenation: *PRPF4*; *SART1*, also known as 110 kDa SR-related protein of the U4/U6.U5 tri-snRNP or as hypoxia associated factor (HAF); and *LSM8*, U6 snRNA-associated Sm-like protein. The 110 kDa SR-related protein was localized in all retinal cells including photoreceptors.

**Conclusions:**

Regulation by changes in oxygenation within the U4/U6.U5 tri-snRP complex could be particularly important for photoreceptors where oxygen consumption follows a circadian rhythm. If the U4/U6.U5 tri-snRP complex is already impaired by mutations in any of the four genes causing RP, it may be unable to follow properly the physiological demands of oxygenation which are mediated by the four hypoxia-regulated proteins emerging in this study. Selective vulnerability may involve complex combinations of widely expressed genes, specific cellular functions and local energy availability.

## Introduction

The retina is well known for its high rate of energy consumption [1]. Oxygen delivery to the photoreceptors in the outer retina differs from that in other nervous tissues, in that oxygen is provided by diffusion from the choroidal circulation, which requires very high levels of blood flow [1,2]. There is no local coupling between metabolic expenditures of photoreceptors and blood flow in the choroidal vessels, and therefore oxygen tension can decrease to low levels at the proximal side of the inner segment in the face of high energy expenditures during dark-adaptation [2]. Furthermore, photoreceptor function is rapidly affected after the onset of hypoxia [2]. The expression of the oxygen-storage molecule, neuroglobin, in the photoreceptor inner segment further underlines the importance of regulation of oxygen availability [3]. Importantly, chronic ischemia-hypoxia of the rat retina, induced by occlusion of both common carotid arteries, can be associated with photoreceptor degeneration [4,5]. We have shown in rats that such photoreceptor degeneration will occurr with a delay of several weeks to months after occlusion of both common carotid arteries [6]. Thus, metabolic failure and oxidative stress may be related to slow degeneration of photoreceptors in rodent models of ocular ischemia [5].

Several studies have suggested that the high rate of oxygen consumption of photoreceptors is involved in the vulnerability to retinitis pigmentosa (RP) [1,7-11]. Such interaction could be quite general, in that moderately dysfunctional photoreceptors carrying RP mutations in a variety of cell-specific genes could be prone to undergo apoptosis when challenged with transient energetic deficits, e.g., due to changes in membrane function and calcium homeostasis [11]. The link could also be more specific through regulation of gene expression in response to the state of oxygenation. For example, the *RP1* gene was identified as oxygen-regulated photoreceptor protein 1 (ORP1) when retinal gene expression was studied under conditions of hypoxia [12]. Bernstein et al. noted that the expression of a given disease causing gene within the retina may not be selective for the cells affected by degeneration [13]. Furthermore, mutations in ten genes cause retina-specific degeneration although these genes are also expressed in several other organs, i.e., the RP9 gene *PAP1, IMPDH, MERTK, RPGR, RP2, CHM, TIMP3*, and three pre-mRNA splicing factors [14]. Retina-specific splice variants and high expression of the gene in the retina relative to other organs may explain the selective vulnerability in the case of *IMPDH1* mutations in RP10 [15,16]. Selective expression of a critical binding partner in the retina is another possible scenario [16]. In this paper, we postulate that widely expressed genes causing RP may be components of protein complexes whose function is dependent on oxygenation through regulation of gene expression and that the cyclical character of oxygenation in the outer retina introduces a selective deleterious effect on photoreceptors. Thereby, we will primarily use the term “hypoxia” to indicate a critically low level of oxygenation, whereas abnormally high levels of oxygen and oxidative stress may as well be involved [10].

Selective degeneration of photoreceptor cells is found in autosomal dominant retinitis pigmentosa (ADRP) associated with mutations in genes coding for proteins involved in pre-mRNA splicing in the spliceosome. This selectivity for the retina is striking, because the genes involved are house keeping genes [14,16]. RP11 (OMIM 600138) is associated with mutations in precursor mRNA-processing factor 31 (*PRPF31*) [17-19]; RP13 (OMIM 600059) with precursor mRNA-processing factor 8 (*PRPF8*) mutations [20]; and RP18 (OMIM 601414) with precursor mRNA-processing factor 3 (*PRPF3*) mutations [21]. PRPF31 and PRPF3 are protein components of the U4/U6 small nuclear ribonucleoprotein (snRNP) complex, and PRPF8 is the core component of the U5 snRNP [14,16]. Additionally, although the evidence is not as strong, mutations in Pim1 kinase associated protein (*PAP1*) were found to cause RP9 (OMIM 607331) [22]. PAP1 has also been characterized as a component of the U4/U6.U5 tri-snRNP complex, which interacts with PRPF3 [23,24]. These four proteins (PRPF31, PRPF8, PRPF3, PAP1) contribute to the formation and function of the U4/U6.U5 tri-snRNP complex, which involves at least thirty proteins [14,16]. Although pre-mRNA splicing should be important for all cells in the body, only photoreceptor cell death occurs in these four forms of ADRP. There is no evidence in the literature that retina-specific splice variants exist for these genes. Haploinsufficiency has been proposed as the pathogenic factor in ADRP [16,18], especially for *PRPF31* (RP11) [17,25-27]. One scenario for the selective vulnerability of photoreceptors is quantitative, the regulation of splicing is necessary for photoreceptors because the levels of mRNA for rhodopsin (and perhaps other genes) undergo considerable change due to the circadian rhythm [18,21,26]. Recently, photoreceptor genes affected by *PRPF31* mutations have been reported [28]. Other studies have shown changes in nuclear trafficking caused by a reduced solubility of mutant *PRPF31* [29]. Mutant PRPF3 associated with RP differed from the wild type protein by forming abnormally big protein aggregates in transfected photoreceptor cells, and aggregation of mutant *PRPF3* inside the nucleus triggered apoptosis in photoreceptor cells [30]. Yeast two-hybrid analyses have suggested a link between RP and an aberrant hPrp31-hPrp6 interaction that blocks U4/U6-U5 tri-snRNP formation [31]. In the present study, we examined the hypothesis that the function of the U4/U6.U5 tri-snRNP complex is oxygen regulated because of the energy dependence of splicing [32], and that this mechanism provides specificity for the effects of the mutation on the outer retina.

Using a theoretical approach, we have previously explored links between the genetics of nervous system disorders and oxygen regulation of gene expression [33]. We compiled a listing of genes regulated by ischemia-hypoxia in the rodent brain from a detailed evaluation of microarray studies and the original literature, and correlated this list with a set of candidate genes for schizophrenia [33]. At present, the information for gene expression changes in ischemia-hypoxia in the retina [34] is too limited to perform a similar analysis for genetic disorders of the retina. It has been proposed that gene expression related to fundamental pathological events in the brain and retina should be sufficiently similar, so that data collections can be carried over from the brain to the retina in a theoretical approach [35]. Such strategy could be especially fruitful for globally expressed RP genes as discussed in the preceding text. In this paper, we used the brain-based database and literature searches to look for potential connections between globally expressed RP genes and changes in oxygenation with a specific focus on pre-mRNA splicing. We identified four genes involved in the U4/U6.U5 tri-snRNP of the spliceosome during this search. Databases for retinal gene expression were then used to confirm the expression of these genes. Finally, immunohistochemistry was used to identify the protein expressed by one of the four genes in the monkey and human retina.

## Methods

### Stage A

We built a database of 24 published gene expression studies in brain ischemia-hypoxia, starting with our cDNA microarray study of focal brain ischemia [36]. Gene lists were transformed into an Excel (Microsoft) datasheet, and genes were identified by running clone identification numbers for individual probes through Nucleotide, UniGene, and Entrez Gene (NCBI). Selected probes of the microarray analysis were verified by using BLAST searches (Nucleotide, NCBI). We generated an ischemia-hypoxia response (IHR) gene list after sorting by gene symbol and removing redundancy. Information for individual genes was retrieved from OMIM (NCBI) and the literature (PubMed, NCBI). Genes were collected under different categories that were defined by mechanisms of ischemic-hypoxic cell damage and neurodegeneration. Two studies relevant to hypoxia regulation in neurons by hypoxia inducible factor 1 and 2 (HIF-1, HIF-2) were considered in addition [37,38]. Information for gene expression studies in retinal ischemia was searched using PubMed, but only one data set [34] was found to be in parallel with the IHR gene list. We also reprobed the original data set of our microarray study [36] for specific genes encountered during the search.

### Stage B

UniGene and PubMed searches were used to test whether widely expressed RP genes were also found in the brain. A listing of RP genes with widespread expression and function were taken from the review of Pacione et al. [14]: *RP9/PAP1, IMPDH1, MERTK, RPGR, RP2, CHM, TIMP3*, and three pre-mRNA splicing factors. An update was performed, using database searches the Retinal Information Network (RetNet) and the Retina International Scientific Newsletter (RISN), which led to the addition of *SEMA4A* [39] and *CA4* [40]. The analysis of brain expression aimed at globally expressed genes involved in RP and was not designed as a comprehensive study of brain expression of all RP genes.

### Stage C

The IHR gene list was then searched for genes involved in RP and related retinal dystrophies. Gene symbols were run through OMIM to see whether a role in specific ophthalmic diseases was listed or if the gene was under consideration as a risk gene. Lists in RetNet and RISN were regularly surveyed for matches to ischemia-hypoxia regulated genes. Several retina-specific disease genes were found oxygen regulated, and one RP-interacting gene involved in pre-mRNA splicing (RP9 see Results) was retrieved.

### Stage D

We then focused on genes related to pre-mRNA splicing in the IHR gene list, because of the unique overpresentation of genes of the U4/U6.U5 tri-snRNP complex among widely expressed RP genes [14], the expression of these genes in the brain as documented at Stage B, and the finding of one gene related to pre-mRNA splicing and RP at Stage C (see Results). IHR genes related to mRNA processing were identified, and specific links to the U4/U6.U5 tri-snRNP were explored using information available through Entrez Gene, OMIM, and PubMed. The purpose of this analysis was to identify relations between the hypoxia regulation and pre-mRNA splicing, i.e., oxygen dependence of genes interacting with the RP genes in the spliceosome.

### Stage E

To analyze the expression of genes identified during Stage D in the human retina, a database search for mRNA in NEIBank at the National Eye Institute was carried out. Gene expression profiles in the mouse retina were examined using serial analysis of gene expression (SAGE) derived databases [41]. The EyeBrowse (Browse) database was also used. This database provides an alignment of eye specific ESTs with the human genome sequence according to the Genome Browser (UCSC). Studies with immunohistochemistry for a selected protein are described in Stage H.

### Stage F

Bioinformatics were used to identify target genes regulated by HAF (i.e., *SART1*), which is one of the oxygenation-regulated members of the U4/U6.U5 tri-snRNP complex (see Results). The EP17 consensus sequence (5′-CCC CCA CCC CCA CCC GC-3′) for HAF binding in the promoter of the *EPO* gene [42] was used in a BLAST (NCBI) search for “short nearly exact matches.” Only fully annotated genes and hits on putative regulatory regions were evaluated. Published information was retrieved to connect such genes to retinal function and pathology.

### Stage G

This study was conducted after approval of a protocol by the Animal Care and Use Committee and Review Board for Animal Research of the University of Miami and conformed to the Statement for the Use of Animals in Ophthalmic and Vision Research by the Association for Research in Vision and Ophthalmology (ARVO). Whole eye globes were provided in accordance with a university-wide ACUC approved Tissue Sharing Protocol. Whole eyes of two year old, healthy, male cynomolgus monkeys (*Macaca fascicularis*) were received immediately after euthanasia and the posterior pole was dissected less than 2 h postmortem, immediately fixed by immersion in 10% buffered formalin for 16 h, and then transferred to phosphate buffered saline (PBS). The retinas of n=3 monkeys were processed the immunohistochemical study (supplementing stage E of the analysis). The tissue were embedded in paraffin, and sections were cut with a microtome to 8 μm thickness, then mounted on glass slides coated with poly-L-lysine (Sigma-Aldrich, St. Louis, MO). Immunohistochemistry was performed employing the avidin-biotin (ABC) technique using antigen retrieval with microwave treatment [6]. Sections were rinsed with PBS, then incubated in 0.3% H_2_O_2_ for 15 min to inactivate endogenous peroxidase and then blocked with 10% normal goat serum, 0.25% Triton X-100, and Avidin D solution (Avidin/Biotin blocking kit, Vector, Burlingame, CA) for 30 min. Next sections were washed with 0.25% Triton X-100 in PBS and incubated in PBS containing 0.25% of Triton X-100, biotin solution (avidin/biotin blocking kit, Vector), 1% normal goat serum, and the primary antibody for 1 h at room temperature. Affinity-purified rabbit antibodies to an N-terminal sequence of the 110 kDa SR-related protein of the U4/U6.U5 tri-snRNP were kindly provided by Drs. Olga V. Makarova and Evgeny M. Makarov, and Prof. R. Luehrmann, Department of Cellular Biochemistry, Max-Planck-Institute for Biophysical Chemistry, Goettingen, Germany [43]. Antibodies were applied at a dilution of 1:200. Sections were washed with 0.25% Triton X-100 in PBS and treated with biotin-conjugated goat anti-rabbit IgG (1:250; BA-1000; Vector) followed by streptavidin-HRP (K0377; Dako, Carpintera, CA). Diaminobenzidine (0.05%) including 0.003% H_2_O_2_ was used for the permanent localization of peroxidase activity. Omission of the primary antibody and absorption with the native immunogenic peptide served as control. Sections were dehydrated in a series of alcohols and xylene, and coverslipped with DePeX (Aldrich, Milwaukee, WI). Some sections were counterstained with hematoxylin to visualize the nucleus.

Retinal specimens from a 46 year old female donor became available (University of Regensburg, Germany) within 1 h after enucleation due to severe herpes simplex keratitis. The tissue was fixed in 4% paraformaldehyde in PBS (pH 7.4) overnight at 4 °C. Specimens were embedded in paraffin, and sections were cut at 2 μm with a microtome, then fixed on Superfrost® plus glass slides (Menzel, Braunschweig, Germany). Immunohistochemistry was performed with a modified ABC technique, using antigen retrieval with microwave treatment. Sections were incubated in citric acid/citrate buffer (0.2 mM citric acid, 9.8 mM sodium citrate, pH 7.3) for 2 min at 800 W and then 10 min at 240 W in a microwave oven. The rabbit antibody to an N-terminal sequence of the 110 kDa SR-related protein of the U4/U6.U5 tri-snRNP was applied in a 1:200 dilution and incubated using Shandon Coverplates (Thermo, Pittsburgh, PA) and the DakoCytomation LSAB2 System-HRP (K0672, DakoCytomation, Carpinteria, CA) according to the manufacturers' instructions. Primary antibody absorbed with the native peptide served as control. Sections were dehydrated and coverslipped with Entellan new (Merck, Darmstadt, Germany).

### Stage H

Based on the matches found for pre-mRNA splicing (see Results), it was considered whether other RP forms may involve genes related to pre-mRNA splicing. Genes identified at Stage D were analyzed. Human, mice and dog gene loci were retrieved from the Genome Browser and OMIM. Chromosomal localizations were checked in the RISN for related retinal dystrophies and for RP forms in which the underlying gene has not yet been identified. The literature [44,45] and the RISN animal database were searched for mice and dog retinal disorders.

### Stage I

Several studies have stressed the importance of oxidative stress for photoreceptors in RP [5,10]. The IHR database was built upon experiments using recirculation and reoxygenation of the brain after ischemia, which leads to oxidative stress. If genes related to oxidative stress are an intrinsic part of the database, correlations with retinal genes may arise from oxidative stress. To estimate this contribution, an additional database specific for the genomic response to oxidative stress was generated based on the transcription factor nuclear factor erythroid-derived 2-like 2 (NRF2) [46]. NRF2 mediates the response to oxidative stress by binding to the antioxidant response element (ARE). Wang et al. recently provided databases of NRF2-target genes [46]. The NRF2-ARE related databases were then scanned for retinal disorders (as in Stage C) and for genes related to pre-mRNA splicing (as in Stage D).

## Results

### Stage A: Database of ischemia-hypoxia response genes of the brain

About 2,500 gene entries were collected from 24 microarray studies of ischemia-hypoxia in the brain. Using the clone ID number, we found about 1,750 genes could be linked to a gene symbol, forming the IHR gene list. Of these, 78% genes were only found once and 22% were found two or more times. The IHR genes made up 7% of all genes. The genes listed in a study of retinal ischemia [34] overlapped by about 40% with the genes in the IHR gene list obtained in the brain. This comparison provides proof of principle that many genes studied in the brain are likely to be involved in the retina under conditions of abnormal oxygenation [35].

### Stage B: Brain expression of ubiquitously expressed *retinitis pigmentosa* genes

The expression profile of the RP genes with global expression listed in the review of Pacione et al. [14] and of two additional genes (*CA4* [39], *SEMA4A* [40]) was examined by using transcriptome information from UniGene. Expression in the brain was listed for *RP9/PAP1, IMPDH1, MERTK, RPGR, RP2, CHM, TIMP3, PRPF3, PRPF8, PRPF31, SEMA4A* and *CA4*. Thus, a database established in the brain is suitable to explore features of regulation for these RP genes.

### Stage C: Ischemia-hypoxia response genes related to *retinitis pigmentosa* and other retinal dystrophies

When probing the IHR gene list, several genes related to RP and retinal dystrophies presented as ischemia-hypoxia regulated. *CNGB1* is mutated in autosomal recessive retinitis pigmentosa (ARRP, OMIM 600724) and is expressed in the brain (UniGene Hs.147062), where it was found regulated in ischemia [47]. Mutations of *SEMA4A* were found in RP35 (OMIM 610282) and in dominant cone-rod dystrophy (CRD; listed in RISN) [39], *SEMA4A* is ischemia regulated in the brain [48]. *RRG4* mRNA (*UNC119 - HRG4*) was found down-regulated after neonatal brain ischemia/hypoxia [49], and mutations in this gene cause a rare form of cone-rod dystrophy - CRD (OMIM 604011) [50]. *SOX2* mutations cause anophthalmia (OMIM 184429) and brain malformations [51,52]. *Sox2* is down-regulated in brain ischemia [48].

### Stage D: Ischemia-hypoxia response genes in the U4/U6.U5 tri-snRNP complex

Our analysis focused on genes involved in pre-mRNA splicing due to the unique situation that four RP genes with global expression contribute to one functional protein complex. Hypoxia regulation of genes interacting with RP genes within the same protein complex may exert an indirect effect of oxygenation. There were 37 genes related to mRNA processing on the IHR gene list and in publications on HIF targets in neurons [37,38]. When gene products were examined for a specific role in the U4/U6.U5 tri-snRNP complex, four genes were identified. A complete expression listing of genes from brain involved in mRNA processing or under hypoxia regulation is still not not available. Therefore, an enrichment of hypoxia controlled genes among the mRNA processing genes cannot be accurately determined. The following estimate can be made. Based on data by Pacione et al. [14], 30 genes out of all genes (ca. 28,000) code for the protein components of the U4/U6.U5 tri-snRNP complex. Thus, among the n=1,750 genes of IHR gene database, two genes could be related to the U4/U6.U5 tri-snRNP complex by chance, whereas four genes were observed.

The first gene was precursor mRNA-processing factor 4 (*PRPF4*) which was found upregulated in global brain ischemia [53]. The presence of PRPF4 in U4/U6.U5 tri-snRNPs has been described whereby PRPF4 was tightly associated with U4/U6 snRNP [54,55]. PRPF4 binds to the central region of PRPF3 (causing RP18), and PRPF3 may recruit PRPF4 for the U4/U6 snRNP assembly [56].

The second gene found was *SART1*, human squamous cell carcinoma antigen recognized by T cells 1 [57]. This gene was reported to be down-regulated in a rat model of neonatal ischemia/hypoxia of the brain [49]. Human *SART1* (GeneID 9092; OMIM 605941) has been also characterized as the 110 kDa SR-related protein of the U4/U6.U5 tri-snRNP [43], which is critically important for the recruitment of the U4/U6.U5 tri-snRNP complex to the pre-spliceosome [43]. Additional information for *SART1* and a role in hypoxia was found. The original data set of our cDNA microarray study in brain ischemia [36] showed a trend for down-regulation of the mouse *Sart1* mRNA (−1.5) that was measured with the probe sequence AA607769 (Nucleotide, NCBI). A BLAST search of the probe sequence (AA607769) showed 97% identity with clone AF129931.1 which was listed as “mus musculus hypoxia associated factor (Haf)” while being linked to *Sart1* in GeneID 20227 (Entrez Gene, NCBI). By following the term “hypoxia associated factor (Haf)” in the original literature, we found HAF was originally characterized during the analysis of the hypoxia induced expression of EPO and vascular endothelial growth factor (VEGF) under hypoxic conditions [42,58].

The third gene was *LSM8*, U6 snRNA-associated Sm-like protein, which was down-regulated in focal ischemia in mice [48]. Achsel et al. [59] showed that the so-called LSm proteins (which are related to the “Sm” proteins binding to the Sm site of small nuclear RNAs) facilitate the formation of U4/U6 duplexes.

The fourth gene was *Pim1*, which was found to be strongly upregulated in an extended analysis of our microarray study [36]. Pim1 binds to PAP1 [60], the gene carrying mutations in RP9 [22], which, in turn, contributes to the U4/U6.U5 tri-snRNP complex [23,24].

### Stage E: Expression information for selected genes

Homo sapiens *PRPF4* mRNA was found in the NEI “human retina non-normalized” database (NbLib0042); and clone XM_131444 was linked to *Prpf4* in the “dbEST mouse retina (non-normalized) data collection” (NbLib0027). Expression of *SART1* (clone AF353625) was detected in the dbEST human retina list. Gene expression profiling using SAGE provided evidence for *Sart1* mRNA expression in the mouse retina and in the microdissected outer nuclear layer [41]. Expression of *LSM8* mRNA was listed for the eye in UniGene (Hs.446179) but not in NEIBank. A subsequent search in EyeBrowse showed an alignment of eye ESTs to the human genome sequence for *PRPF4* (retina, BQ638822), *SART1* (retina, W27222; RPE, BM694274), and *PIM1* (RPE, CA390708).

### Stage F: Putative target genes for SART1/HAF

HAF regulation has been shown for *EPO* and *VEGF* [42,58]. These genes are involved in retinal pathology. Therefore, additional genes regulated by HAF could be relevant to retinal function and pathology. Targets of SART1/HAF regulation were trapped using the EP17 consensus sequence for HAF binding in the promoter of the EPO gene [42] in a BLAST search for “short nearly exact matches.” Ten annotated genes had matches in putative regulatory regions (i.e., outside protein coding sequences). Two of these putative HAF target genes were of interest for retinal degenerations: (a) Mm. ATPase, Na^+^/K+ transporting, beta 2 polypeptide (*Atp1b2*) was known originally as AMOG-glial cell adhesion molecule (16/17 matches, NM_031415, 3′ UTR), and disruption of *Amog* in mice leads to apoptotic cell death of photoreceptors [61]; and (b) Neuroblastoma, suppression of tumorigenicity (*Nbl1*, “DAN”; 16/17 matches, NM_008675, 3′ UTR) is known to be regulated by brain ischemia and is expressed in the eye.

### Stage G: Immunohistochemical study

**Figure 1:**
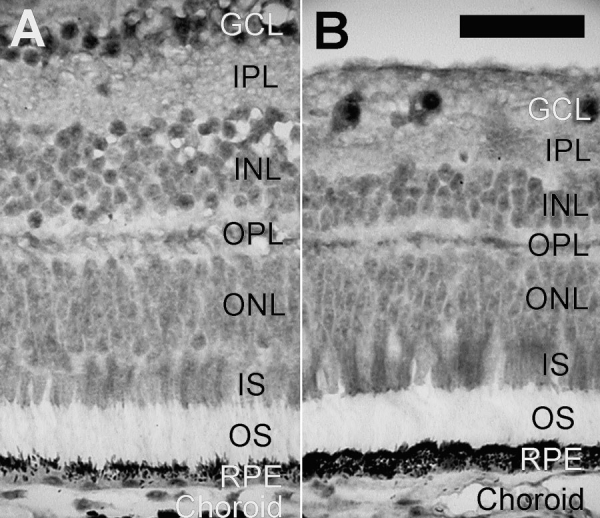
Immunostaining for 110 kDa SR-related protein in cynomolgus monkey retina. Immunohistochemical detection of the 110 kDa SR-related protein of the U4/U6.U5 tri-snRNP (hypoxia-associated factor) in paraffin sections of the cynomolgus monkey retina. **A**: View of the central retina; **B**: view of the peripheral retina. Note that the dark signal in the retinal pigment epithelium (RPE) and choroid derives from the melanin. These areas were included in the image to illustrate the total absence of specific immunolabeling from the outer segments. The following abbreviations were used in this figure: ganglion cell layer (GCL); inner nuclear layer (INL); inner plexiform layer (IPL); inner segments of photoreceptors (IS); outer nuclear layer (ONL); outer segments of photoreceptors (OS); retinal pigment epithelium (RPE). The calibration bar is equal to 40 μm.

**Figure 2:**
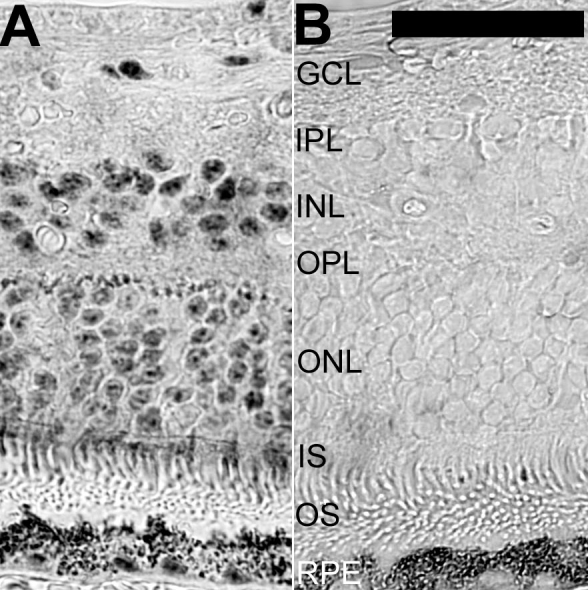
Immunostaining for 110 kDa SR-related protein in human retina. Immunohistochemical detection of the 110 kDa SR-related protein of the U4/U6.U5 tri-snRNP (hypoxia-associated factor) was carried out in paraffin sections of the peripheral human retina. The dark signal in the retinal pigment epithelium (RPE) and choroid derives from the melanin. These areas were included in the image to illustrate the total absence of specific immunolabeling from the outer segments. **A**: Distinct cellular labeling was obtained with the antibody to 110 kDa SR-related protein of the U4/U6.U5 tri-snRNP. **B**: Immunoreactivity was absent after absorption of the primary antibody with immunogenic peptides. The following abbreviations were used in this figure: ganglion cell layer (GCL), inner nuclear layer (INL), inner plexiform layer (IPL), outer plexiform layer (OPL), inner segments of photoreceptors (IS), outer nuclear layer (ONL), and outer segments of photoreceptors (OS). The calibration bar is equal to 40 μm.

Immunoreactivity for the 110 kDa SR-related protein was found in all retinal cells of the cynomolgus monkey (Figure 1). Strong labeling with a fine granular pattern was obtained in the nucleus of retinal ganglion cells (RGCs). The cytoplasm of large RGCs also showed immunoreactivity. Nuclei of neurons of the inner nuclear layer were positive. Strong expression was found in the nuclei of photoreceptors and in the inner segments, whereas the outer segments were negative. Nuclei of the retinal pigment epithelium (RPE), vascular cells, and optic nerve glial cells were also labeled. Specific labeling was absent after omission of the primary antibody and after absorption of the primary antibody with the native peptide. Subsequently, sections of a human retina were labeled, using the ABC technique for detection (Figure 2). The staining pattern obtained in the human retina was identical to that seen for the monkey retina. Staining of the nucleus was seen for RGCs and cells in the inner nuclear layer. The nuclei and the inner segments of photoreceptors showed immunoreactivity whereas the outer segments were unlabeled. The RPE and choroidal vessels could not be evaluated due to the presence of pigment. All immunostaining was absent in absorption controls. A fine band of labeling was seen in the OPL of the primate and human retinas (Figure 1 and Figure 2), which remains unexplained, because generalized staining indicative of expression in synapses or other small organelles was not found. This raises the question whether splicing proteins can be localized to the specialized dendrites of the axonless horizontal cells in OPL.

### Stage H: Links between splicing and additional forms of retinitis pigmentosa

Several RP genes remain to be identified. The loci for the hypoxia-regulated genes related to the U4/U6.U5 tri-snRNP complex by our analysis (*PRPF4, SART/HAF1, LSM8*, *PIM1*) were scanned in OMIM and RetNet. *SART1/HAF* is on 11q13, where a locus for the autosomal dominant neovascular inflammatory vitreoretinopathy (VRNI) was mapped (OMIM 193235). VRNI is a blinding disorder that presents with some clinical features of RP. The mouse or dog orthologs did not map to loci containing novel retinal disorders.

### Stage I: Exploring a role for gene regulation by oxidative stress

Since several studies have stressed the importance of oxidative stress for photoreceptors in RP [5,10], the databases provided by Wang et al. [46] for the genomic response to oxidative stress were examined. Combining the data provided by Wang et. (supplemental tables 2 and 3 in Wang et. paper) [46]; n=674 putative putative NRF2- target genes were available for analysis which were scanned for genes related to retinal disorders. Matches were found for *CACNA1F* (Night Blindness, congenital, stationary, X-linked, Type 2- CSNB2; OMIM 300071), *GNAT1* (Night Blindness, congenital, stationary, autosomal dominant 3-CSNBAD3; OMIM 610444), *RHO* (Retinitis pigmentosa 4-RP4; OMIM 180380; also, Night Blindness, congenital, stationary, autosomal dominant 1-CSNBAD1; OMIM 610445), and *OAT* (Gyrate atrophy; OMIM 258870). We reencountered *PIM1* and *SEMA4A*, suggesting a role of oxidative stress for the regulation of these two matched IHR genes. When looking for additional genes related to the U4/U6.U5-tri-SNP, no genes were found in the list of fully annotated NRF2 target genes (n=412) that were provided by Wang et al. (supplemetal table 2 in Wang et. paper) [46]. This negative finding indirectly suggests that the correlations found at stage D are due to a hypoxic component which is in line with the original hypothesis.

## Discussion

Cyclical changes between light and dark are associated with considerable physiological changes in oxygen levels at the level of the outer retina [1,2]. Therefore, excessive metabolic stress in photoreceptors could be expected to create specific localized problems resulting in RP forms that involve widely expressed genes, e.g., genes involved in dysfunction of pre-mRNA splicing. To estimate which genes could be regulated by variable oxygenation in the retina, we applied a comparative analysis of gene expression under experimental ischemic-hypoxic conditions in the brain, based on the hypothesis that similar genes are regulated in the brain and retina. The hypothesis was supported by the similarity between changes seen in the brain and the retina after ischemia [6] and by the evaluation of gene expression profiles in one study on the ischemic retina [34]. Several genes related to RNA processing turned out to be regulated after ischemia-hypoxia in the brain, and, four genes could be specifically related to the U4/U6.U5 tri-snRNP. Database searches showed that these genes were also likely to be expressed in the retina.

The first gene found was *PRPF4*. The PRPF4 protein binds to the central region of PRPF3. Because mutations in *PRPF3* cause RP18 [21] and PRPF3 interacts with PAP1 (causing RP9), a strong hypothetical link to ischemia-hypoxia (oxygenation) involvement in ADRP emerged from the present analysis. The second IHR gene was the 110 kDa SR-related protein of the U4/U6.U5 tri-snRNP, also known as *SART1* or *HAF* [42,43,57]. Immunohistochemistry verified expression in photoreceptors of the primate and human retina. Dual functions of proteins in transcriptional regulation and pre-mRNA splicing have been recognized [62]. Accordingly, the 110 kDa SR-related protein is essential for the recruitment of the U4/U6.U5 tri-snRNP complex to the spliceosome [43], and the same protein plays a role in the regulation of expression of *EPO* and *VEGF* by hypoxia under the name of HAF [42,58]. Intriguingly, EPO showed protective functions for photoreceptors [63]. In the photoreceptor layer, intrinsic physiological changes in oxygenation [2] could control the activity of the SART1/HAF which in turn affects the expression as well as the splicing of specific target genes, e.g., *EPO*, *VEGF*, *Atp1b2*, and *Nbl1*. The *LSM8* gene can be also set in the context of the U6 complex [59]. The fourth gene found regulated in the brain, *PIM1*, targets PAP1 (causing RP9) for phosphorylation in vitro [60]. A 2004 study showed that the interaction between PAP1 and PIM1 does not play a major role in vivo [23], so it remains to be seen whether PAP1 is indirectly regulated by oxygenation.

Regulation of *PRPF4*, *SART1*/*HAF*, *LSM8, and PIM1* by changes in oxygenation argues for a reversible functional change within the U4/U6.U5 tri-snRP complex, which could be especially important for photoreceptors where oxygenation and oxygen consumption follow a circadian rhythm. Mutations in any of the four ADRP genes involved in the assembly of the U4/U6.U5 tri-snRP complex, i.e., *PRPF31* (RP11), *PRPF8* (RP13), *PRPF3* (RP18), and *PAP1* (RP9), may influence either the ability to undergo this change or its reversibility. If the U4/U6.U5 tri-snRP complex is already impaired by mutations in any of the four genes causing ADRP, it may be unable to deal with the physiological changes of oxygenation which are mediated by the four hypoxia-regulated proteins emerging in this study. A dominant negative effect is not supported by this mechanism as well not by the late onset of ADRP in the patients. Therefore, a reduced function of the U4/U6.U5 tri-snRP complex under hypoxic conditions will cause an alternating haploinsufficiency during the diurnal rhythm, which exerts a slow, persisting damage to the photoreceptor cells, resulting in late onset disease. However, it should be noted that the correlations found here cannot be taken to indicate an exclusive importance of hypoxia for the U4/U6.U5 tri-snRNP as compared to other protein complexes. Analysis of other components of the spliceosome may be fruitful.

Oxidative stress has been considered to be a major factor in retinal degeneration [5,10]. Our analysis has stressed “hypoxia” throughout, but it should be noted that hypoxia and oxidative stress are closely associated. It has been argued that photoreceptors are optimally stable in hypoxia, and are destabilized by both hypoxia and hyperoxia [64].

In fact, the IHR gene database contains multiple genes from the brain subjected to reoxygenation when profound oxidative stress occurs [36]. We surveyed databases from a recent bioinformatics study [46] to probe for a specific contribution of antioxidant genes, whose function is to counteract oxidative stress. *PIM1* was the only gene with a role in pre-mRNA splicing found to match. This comparison renders it more likely that hypoxia and metabolic stress were involved. There is an oxygen sink on the promixal side on the inner segment and outer nuclear layer where the gene products of the ADRPs related to pre-mRNA splicing should play important roles [2]. Furthermore, the initial loss of photoreceptors in RP may lead to an alleviation of the physiological hypoxia and relative hyperoxia [65].

More than 40% of cases of ADRP could not be linked to known mutations [66], and novel genes may also relate to pre-mRNA splicing. For the RP33 locus, *ASCC3L1*, also known as U5 snRNP-specific protein, was already suggested as a candidate gene [67].

Interestingly, known RP genes may play a larger role in the nucleus than is presently recognized. The (recessive) RP26 gene encodes ceramide kinase-like protein (*CERKL*) which was linked to nuclear functions. As noted by Inagaki et al. [68] proteins encoded by (dominant or recessive) RP1 and (recessive) RP14 are known to be localized to the nucleolus [68]. Yoon and colleagues showed that the *RP2* gene product undergoes re-localization into the nucleus upon treatment of cells with DNA damaging agents inducing oxidative stress, most notably solar simulated light and UVA radiation, and proposed a previously unrecognized role as a DNA damage response factor and 3′ to 5′ exonuclease [69]. Inosine monophosphate dehydrogenase type I (*IMPDH1*) mutations in RP10 (OMIM 146690) may influence the binding of the protein to single stranded nucleic acids [70].

The concept of an influence of oxygenation on gene expression and function of a protein machinery is only one of several explanations for selective vulnerability of photoreceptors in RP forms that involve widely expressed RP genes. Other possible explanations include strong differences in expression across different organs or even the presence of retina-specific splice variants [16]. Nevertheless, changes in gene expression and function due to strong local changes in oxygenation may relate to the clinically variable and delayed manifestation of ADRP. Hypoxia has already been implicated in the variable clinical manifestation of *RP1* [14,71]. Two of the ADRP forms related to pre-mRNA splicing show variable clinical expression, which could hint at a gene-environment interaction. Large variations in retinal dysfunction across family members with RP9 have been reported [14,72], and RP11 caused by *PRPF31* mutations shows generation skipping [26,27,66]. It will be important to examine the critical threshold concentrations for the respective proteins. Based on the findings in this paper, we feel there is a need for further studies of genes related to the U4/U6.U5 tri-snRNP, using retinal tissue under ischemic-hypoxic conditions.

## References

[1] Yu DY, Cringle SJ (2005). Retinal degeneration and local oxygen metabolism.. Exp Eye Res.

[2] Wangsa-Wirawan ND, Linsenmeier RA (2003). Retinal oxygen: fundamental and clinical aspects.. Arch Ophthalmol.

[3] Schmidt M, Giessl A, Laufs T, Hankeln T, Wolfrum U, Burmester T (2003). How does the eye breathe? Evidence for neuroglobin-mediated oxygen supply in the mammalian retina.. J Biol Chem.

[4] Osborne NN, Safa R, Nash MS (1999). Photoreceptors are preferentially affected in the rat retina following permanent occlusion of the carotid arteries.. Vision Res.

[5] Osborne NN, Casson RJ, Wood JP, Chidlow G, Graham M, Melena J (2004). Retinal ischemia: mechanisms of damage and potential therapeutic strategies.. Prog Retin Eye Res.

[6] Yamamoto H, Schmidt-Kastner R, Hamasaki DI, Yamamoto H, Parel JM (2006). Complex neurodegeneration in retina following moderate ischemia induced by bilateral common carotid artery occlusion in Wistar rats.. Exp Eye Res.

[7] Steinberg RH (1987). Monitoring communications between photoreceptors and pigment epithelial cells: effects of “mild” systemic hypoxia. Friedenwald lecture.. Invest Ophthalmol Vis Sci.

[8] Maslim J, Valter K, Egensperger R, Hollander H, Stone J (1997). Tissue oxygen during a critical developmental period controls the death and survival of photoreceptors.. Invest Ophthalmol Vis Sci.

[9] Travis GH (1998). Mechanisms of cell death in the inherited retinal degenerations.. Am J Hum Genet.

[10] Stone J, Maslim J, Valter-Kocsi K, Mervin K, Bowers F, Chu Y, Barnett N, Provis J, Lewis G, Fisher SK, Bisti S, Gargini C, Cervetto L, Merin S, Peer J (1999). Mechanisms of photoreceptor death and survival in mammalian retina.. Prog Retin Eye Res.

[11] Pierce EA (2001). Pathways to photoreceptor cell death in inherited retinal degenerations.. Bioessays.

[12] Pierce EA, Quinn T, Meehan T, McGee TL, Berson EL, Dryja TP (1999). Mutations in a gene encoding a new oxygen-regulated photoreceptor protein cause dominant retinitis pigmentosa.. Nat Genet.

[13] Bernstein SL, Wong P (1998). Regional expression of disease-related genes in human and monkey retina.. Mol Vis.

[14] Pacione LR, Szego MJ, Ikeda S, Nishina PM, McInnes RR (2003). Progress toward understanding the genetic and biochemical mechanisms of inherited photoreceptor degenerations.. Annu Rev Neurosci.

[15] Bowne SJ, Liu Q, Sullivan LS, Zhu J, Spellicy CJ, Rickman CB, Pierce EA, Daiger SP (2006). Why do mutations in the ubiquitously expressed housekeeping gene IMPDH1 cause retina-specific photoreceptor degeneration?. Invest Ophthalmol Vis Sci.

[16] Mordes D, Luo X, Kar A, Kuo D, Xu L, Fushimi K, Yu G, Sternberg P, Wu JY (2006). Pre-mRNA splicing and retinitis pigmentosa.. Mol Vis.

[17] Vithana EN, Abu-Safieh L, Allen MJ, Carey A, Papaioannou M, Chakarova C, Al-Maghtheh M, Ebenezer ND, Willis C, Moore AT, Bird AC, Hunt DM, Bhattacharya SS (2001). A human homolog of yeast pre-mRNA splicing gene, PRP31, underlies autosomal dominant retinitis pigmentosa on chromosome 19q13.4 (RP11).. Mol Cell.

[18] Deery EC, Vithana EN, Newbold RJ, Gallon VA, Bhattacharya SS, Warren MJ, Hunt DM, Wilkie SE (2002). Disease mechanism for retinitis pigmentosa (RP11) caused by mutations in the splicing factor gene PRPF31.. Hum Mol Genet.

[19] Makarova OV, Makarov EM, Liu S, Vornlocher HP, Luhrmann R (2002). Protein 61K, encoded by a gene (PRPF31) linked to autosomal dominant retinitis pigmentosa, is required for U4/U6*U5 tri-snRNP formation and pre-mRNA splicing.. EMBO J.

[20] McKie AB, McHale JC, Keen TJ, Tarttelin EE, Goliath R, van Lith-Verhoeven JJ, Greenberg J, Ramesar RS, Hoyng CB, Cremers FP, Mackey DA, Bhattacharya SS, Bird AC, Markham AF, Inglehearn CF (2001). Mutations in the pre-mRNA splicing factor gene PRPC8 in autosomal dominant retinitis pigmentosa (RP13).. Hum Mol Genet.

[21] Chakarova CF, Hims MM, Bolz H, Abu-Safieh L, Patel RJ, Papaioannou MG, Inglehearn CF, Keen TJ, Willis C, Moore AT, Rosenberg T, Webster AR, Bird AC, Gal A, Hunt D, Vithana EN, Bhattacharya SS (2002). Mutations in HPRP3, a third member of pre-mRNA splicing factor genes, implicated in autosomal dominant retinitis pigmentosa.. Hum Mol Genet.

[22] Keen TJ, Hims MM, McKie AB, Moore AT, Doran RM, Mackey DA, Mansfield DC, Mueller RF, Bhattacharya SS, Bird AC, Markham AF, Inglehearn CF (2002). Mutations in a protein target of the Pim-1 kinase associated with the RP9 form of autosomal dominant retinitis pigmentosa.. Eur J Hum Genet.

[23] Maita H, Kitaura H, Keen TJ, Inglehearn CF, Ariga H, Iguchi-Ariga SM (2004). PAP-1, the mutated gene underlying the RP9 form of dominant retinitis pigmentosa, is a splicing factor.. Exp Cell Res.

[24] Maita H, Kitaura H, Ariga H, Iguchi-Ariga SM (2005). Association of PAP-1 and Prp3p, the products of causative genes of dominant retinitis pigmentosa, in the tri-snRNP complex.. Exp Cell Res.

[25] Abu-Safieh L, Vithana EN, Mantel I, Holder GE, Pelosini L, Bird AC, Bhattacharya SS (2006). A large deletion in the adRP gene PRPF31: evidence that haploinsufficiency is the cause of disease.. Mol Vis.

[26] Rivolta C, McGee TL, Rio Frio T, Jensen RV, Berson EL, Dryja TP (2006). Variation in retinitis pigmentosa-11 (PRPF31 or RP11) gene expression between symptomatic and asymptomatic patients with dominant RP11 mutations.. Hum Mutat.

[27] Sullivan LS, Bowne SJ, Seaman CR, Blanton SH, Lewis RA, Heckenlively JR, Birch DG, Hughbanks-Wheaton D, Daiger SP (2006). Genomic rearrangements of the PRPF31 gene account for 2.5% of autosomal dominant retinitis pigmentosa.. Invest Ophthalmol Vis Sci.

[28] Mordes D, Yuan L, Xu L, Kawada M, Molday RS, Wu JY (2007). Identification of photoreceptor genes affected by PRPF31 mutations associated with autosomal dominant retinitis pigmentosa.. Neurobiol Dis.

[29] Wilkie SE, Morris KJ, Bhattacharya SS, Warren MJ, Hunt DM (2006). . A study of the nuclear trafficking of the splicing factor protein PRPF31 linked to autosomal dominant retinitis pigmentosa (ADRP).. Biochim Biophys Acta.

[30] Comitato A, Spampanato C, Chakarova C, Sanges D, Bhattacharya SS, Marigo V (2007). Mutations in splicing factor PRPF3, causing retinal degeneration, form detrimental aggregates in photoreceptor cells.. Hum Mol Genet.

[31] Liu S, Li P, Dybkov O, Nottrott S, Hartmuth K, Luhrmann R, Carlomagno T, Wahl MC (2007). Binding of the human Prp31 Nop domain to a composite RNA-protein platform in U4 snRNP.. Science.

[32] Kramer A (1996). The structure and function of proteins involved in mammalian pre-mRNA splicing.. Annu Rev Biochem.

[33] Schmidt-Kastner R, van Os J (2006). W M Steinbusch H, Schmitz C. Gene regulation by hypoxia and the neurodevelopmental origin of schizophrenia.. Schizophr Res.

[34] Yoshimura N, Kikuchi T, Kuroiwa S, Gaun S (2003). Differential temporal and spatial expression of immediate early genes in retinal neurons after ischemia-reperfusion injury.. Invest Ophthalmol Vis Sci.

[35] Vazquez-Chona FR, Khan AN, Chan CK, Moore AN, Dash PK, Hernandez MR, Lu L, Chesler EJ, Manly KF, Williams RW, Geisert EE (2005). Genetic networks controlling retinal injury.. Mol Vis.

[36] Schmidt-Kastner R, Zhang B, Belayev L, Khoutorova L, Amin R, Busto R, Ginsberg MD (2002). DNA microarray analysis of cortical gene expression during early recirculation after focal brain ischemia in rat.. Brain Res Mol Brain Res.

[37] Helton R Cui J Scheel JR Ellison JA Ames C Gibson C Blouw B Ouyang L Dragatsis I Zeitlin S Johnson RS Lipton SA Barlow C Brain-specific knock-out of hypoxia-inducible factor-1alpha reduces rather than increases hypoxic-ischemic damage. J Neurosci 2005 25 4099 107Erratum inJ Neurosci 2005May 1125(19)1p following 4888 15843612 10.1523/JNEUROSCI.4555-04.2005 PMC6724950

[38] Ralph GS, Parham S, Lee SR, Beard GL, Craigon MH, Ward N, White JR, Barber RD, Rayner W, Kingsman SM, Mundy CR, Mazarakis ND, Krige D (2004). Identification of potential stroke targets by lentiviral vector mediated overexpression of HIF-1 alpha and HIF-2 alpha in a primary neuronal model of hypoxia.. J Cereb Blood Flow Metab.

[39] Abid A, Ismail M, Mehdi SQ, Khaliq S (2006). Identification of novel mutations in the SEMA4A gene associated with retinal degenerative diseases.. J Med Genet.

[40] Rebello G, Ramesar R, Vorster A, Roberts L, Ehrenreich L, Oppon E, Gama D, Bardien S, Greenberg J, Bonapace G, Waheed A, Shah GN, Sly WS (2004). Apoptosis-inducing signal sequence mutation in carbonic anhydrase IV identified in patients with the RP17 form of retinitis pigmentosa.. Proc Natl Acad Sci USA.

[41] Blackshaw S, Fraioli RE, Furukawa T, Cepko CL (2001). Comprehensive analysis of photoreceptor gene expression and the identification of candidate retinal disease genes.. Cell.

[42] Gupta M, Mungai PT, Goldwasser E (2000). A new transacting factor that modulates hypoxia-induced expression of the erythropoietin gene.. Blood.

[43] Makarova OV, Makarov EM, Luhrmann R (2001). The 65 and 110 kDa SR-related proteins of the U4/U6.U5 tri-snRNP are essential for the assembly of mature spliceosomes.. EMBO J.

[44] Chang B, Hawes NL, Hurd RE, Davisson MT, Nusinowitz S, Heckenlively JR (2002). Retinal degeneration mutants in the mouse.. Vision Res.

[45] Zangerl B, Sun Q, Pillardy J, Johnson JL, Schweitzer PA, Hernandez AG, Liu L, Acland GM, Aguirre GD (2006). Development and characterization of a normalized canine retinal cDNA library for genomic and expression studies.. Invest Ophthalmol Vis Sci.

[46] Wang X, Tomso DJ, Chorley BN, Cho HY, Cheung VG, Kleeberger SR, Bell DA (2007). Identification of polymorphic antioxidant response elements in the human genome.. Hum Mol Genet.

[47] Lu XC, Williams AJ, Yao C, Berti R, Hartings JA, Whipple R, Vahey MT, Polavarapu RG, Woller KL, Tortella FC, Dave JR (2004). Microarray analysis of acute and delayed gene expression profile in rats after focal ischemic brain injury and reperfusion.. J Neurosci Res.

[48] Tang Y, Pacary E, Freret T, Divoux D, Petit E, Schumann-Bard P, Bernaudin M (2006). Effect of hypoxic preconditioning on brain genomic response before and following ischemia in the adult mouse: identification of potential neuroprotective candidates for stroke.. Neurobiol Dis.

[49] Bernaudin M, Tang Y, Reilly M, Petit E, Sharp FR (2002). Brain genomic response following hypoxia and re-oxygenation in the neonatal rat. Identification of genes that might contribute to hypoxia-induced ischemic tolerance.. J Biol Chem.

[50] Kobayashi A, Higashide T, Hamasaki D, Kubota S, Sakuma H, An W, Fujimaki T, McLaren MJ, Weleber RG, Inana G (2000). HRG4 (UNC119) mutation found in cone-rod dystrophy causes retinal degeneration in a transgenic model.. Invest Ophthalmol Vis Sci.

[51] Ragge NK Lorenz B Schneider A Bushby K de Sanctis L de Sanctis U Salt A Collin JR Vivian AJ Free SL Thompson P Williamson KA Sisodiya SM van Heyningen V Fitzpatrick DR SOX2 anophthalmia syndrome. Am J Med Genet A 2005 135 1 7discussion8 15812812 10.1002/ajmg.a.30642

[52] Sisodiya SM, Ragge NK, Cavalleri GL, Hever A, Lorenz B, Schneider A, Williamson KA, Stevens JM, Free SL, Thompson PJ, van Heyningen V, Fitzpatrick DR (2006). Role of SOX2 mutations in human hippocampal malformations and epilepsy.. Epilepsia.

[53] Yakubov E, Gottlieb M, Gil S, Dinerman P, Fuchs P, Yavin E (2004). Overexpression of genes in the CA1 hippocampus region of adult rat following episodes of global ischemia.. Brain Res Mol Brain Res.

[54] Lauber J Plessel G Prehn S Will CL Fabrizio P Groning K Lane WS Luhrmann R The human U4/U6 snRNP contains 60 and 90kD proteins that are structurally homologous to the yeast splicing factors Prp4p and Prp3p. RNA 1997 3 926 41Erratum inRNA 1997 3 1204 6 9257651 PMC1369537

[55] Wang A, Forman-Kay J, Luo Y, Luo M, Chow YH, Plumb J, Friesen JD, Tsui LC, Heng HH, Woolford JL, Hu J (1997). Identification and characterization of human genes encoding Hprp3p and Hprp4p, interacting components of the spliceosome.. Hum Mol Genet.

[56] Gonzalez-Santos JM, Wang A, Jones J, Ushida C, Liu J, Hu J (2002). Central region of the human splicing factor Hprp3p interacts with Hprp4p.. J Biol Chem.

[57] Shichijo S, Nakao M, Imai Y, Takasu H, Kawamoto M, Niiya F, Yang D, Toh Y, Yamana H, Itoh K (1998). A gene encoding antigenic peptides of human squamous cell carcinoma recognized by cytotoxic T lymphocytes.. J Exp Med.

[58] Gupta M, Goldwasser E (1996). The role of the near upstream sequence in hypoxia-induced expression of the erythropoietin gene.. Nucleic Acids Res.

[59] Achsel T, Brahms H, Kastner B, Bachi A, Wilm M, Luhrmann R (1999). A doughnut-shaped heteromer of human Sm-like proteins binds to the 3′-end of U6 snRNA, thereby facilitating U4/U6 duplex formation in vitro.. EMBO J.

[60] Maita H, Harada Y, Nagakubo D, Kitaura H, Ikeda M, Tamai K, Takahashi K, Ariga H, Iguchi-Ariga SM (2000). PAP-1, a novel target protein of phosphorylation by pim-1 kinase.. Eur J Biochem.

[61] Molthagen M, Schachner M, Bartsch U (1996). Apoptotic cell death of photoreceptor cells in mice deficient for the adhesion molecule on glia (AMOG, the beta 2- subunit of the Na, K-ATPase).. J Neurocytol.

[62] Will CL, Luhrmann R (1997). Protein functions in pre-mRNA splicing.. Curr Opin Cell Biol.

[63] Grimm C, Wenzel A, Groszer M, Mayser H, Seeliger M, Samardzija M, Bauer C, Gassmann M, Reme CE (2002). HIF-1-induced erythropoietin in the hypoxic retina protects against light-induced retinal degeneration.. Nat Med.

[64] Wellard J, Lee D, Valter K, Stone J (2005). Photoreceptors in the rat retina are specifically vulnerable to both hypoxia and hyperoxia.. Vis Neurosci.

[65] de Gooyer TE, Stevenson KA, Humphries P, Simpson DA, Curtis TM, Gardiner TA, Stitt AW (2006). Rod photoreceptor loss in Rho−/− mice reduces retinal hypoxia and hypoxia-regulated gene expression.. Invest Ophthalmol Vis Sci.

[66] Sullivan LS, Bowne SJ, Birch DG, Hughbanks-Wheaton D, Heckenlively JR, Lewis RA, Garcia CA, Ruiz RS, Blanton SH, Northrup H, Gire AI, Seaman R, Duzkale H, Spellicy CJ, Zhu J, Shankar SP, Daiger SP (2006). Prevalence of disease-causing mutations in families with autosomal dominant retinitis pigmentosa: a screen of known genes in 200 families.. Invest Ophthalmol Vis Sci.

[67] Zhao C, Lu S, Zhou X, Zhang X, Zhao K, Larsson C (2006). A novel locus (RP33) for autosomal dominant retinitis pigmentosa mapping to chromosomal region 2cen-q12.1.. Hum Genet.

[68] Inagaki Y, Mitsutake S, Igarashi Y (2006). Identification of a nuclear localization signal in the retinitis pigmentosa-mutated RP26 protein, ceramide kinase-like protein.. Biochem Biophys Res Commun.

[69] Yoon JH, Qiu J, Cai S, Chen Y, Cheetham ME, Shen B, Pfeifer GP (2006). The retinitis pigmentosa-mutated RP2 protein exhibits exonuclease activity and translocates to the nucleus in response to DNA damage.. Exp Cell Res.

[70] McLean JE, Hamaguchi N, Belenky P, Mortimer SE, Stanton M, Hedstrom L (2004). Inosine 5′-monophosphate dehydrogenase binds nucleic acids in vitro and in vivo.. Biochem J.

[71] Jacobson SG, Cideciyan AV, Iannaccone A, Weleber RG, Fishman GA, Maguire AM, Affatigato LM, Bennett J, Pierce EA, Danciger M, Farber DB, Stone EM (2000). Disease expression of RP1 mutations causing autosomal dominant retinitis pigmentosa.. Invest Ophthalmol Vis Sci.

[72] Kim RY, Fitzke FW, Moore AT, Jay M, Inglehearn C, Arden GB, Bhattacharya SS, Bird AC (1995). Autosomal dominant retinitis pigmentosa mapping to chromosome 7p exhibits variable expression.. Br J Ophthalmol.

